# Fixation Stability of the Right and Left Eyes Under Binocular and Monocular Viewing Conditions

**DOI:** 10.3390/life15050703

**Published:** 2025-04-26

**Authors:** Jae-Hyeon Noh, Sang-Yeob Kim, Byeong-Yeon Moon, Hyun Gug Cho, Dong-Sik Yu

**Affiliations:** Department of Optometry, Kangwon National University, Samcheok 25949, Republic of Korea; nys3678@kangwon.ac.kr (J.-H.N.); syk@kangwon.ac.kr (S.-Y.K.); bymoon@kangwon.ac.kr (B.-Y.M.); hyung@kangwon.ac.kr (H.G.C.)

**Keywords:** fixation stability, binocular viewing, monocular viewing, phoria, bivariate contour ellipse area (BCEA), dominant eye

## Abstract

Herein, we investigated changes in fixation stability between the right and left eyes during binocular and monocular viewing in young adults without strabismic binocular vision disorders. Fixation stability was assessed using the bivariate contour ellipse area (BCEA) in 34 healthy participants (15 males, 19 females) in their 20s. Eye-tracking was performed under three conditions: binocular viewing, monocular viewing, and monocular occlusion. Under these conditions, the median BCEA (deg^2^) values for the right and left eyes were 0.95 and 0.75, 1.07 and 0.86, and 1.62 and 1.32, respectively. Fixation stability did not differ significantly between the right and left eyes under the same viewing conditions. However, the left eye demonstrated greater fixation stability under binocular viewing (closed loop/both eyes open) than the right eye under monocular viewing (open loop/one eye closed). Participants with a dominant left eye in binocular viewing had higher fixation stability than those with a dominant right eye. During monocular occlusion, the nondominant eye showed better fixation stability than the dominant eye. A significant quantitative correlation was observed between phoria and fixation stability in the left eye during binocular viewing. These findings show that fixation stability changes with different viewing conditions and is affected by which eye is covered. Therefore, when conducting tests such as ocular alignment, which involve covering one eye, it may be helpful to assess both right and left eye occlusion for ensuring a more comprehensive analysis.

## 1. Introduction

Fixation stability, along with saccadic and pursuit functions, is a key factor in evaluating ocular movements [1,2,3]. Clinically, fixation stability refers to the ability of the eye to maintain a steady gaze on a target. Disorders such as macular disease [4], amblyopia [5,6], strabismus [7,8], anisometropia [9], and neurological dysfunction [10] can compromise fixation stability, leading to impaired visual function. Advanced devices such as microperimeters [11,12] and eye trackers [13,14] have allowed for more precise evaluation of fixation stability, revealing impairments in both binocular coordination and monocular-independent eye movements [15,16,17] in individuals with these disorders. As a result, fixation stability assessment has become an essential tool in ophthalmological and neurological examinations [18]. Fixation stability is not only fundamental to ocular health but also contributes to a better understanding of eye movement dynamics, binocular coordination, and interactions within the oculomotor system. Furthermore, it can provide crucial insights into the detection of abnormalities associated with motor and neurological disorders such as Parkinson’s disease [19]. Therefore, the objective assessment of fixation stability using eye tracking offers valuable implications for both clinical and research settings.

Eye trackers, which are precise and non-invasive, enable detailed analysis of fixation stability, including micro-eye movements. They can assess fixation stability in each eye separately or evaluate binocular coordination [20,21], making them particularly valuable for detecting subtle fixation disturbances. In previous studies [22,23,24,25], we utilized an eye tracker to conduct a more detailed analysis of micro-eye movements in individuals with non-strabismic binocular vision disorders such as phoria, which may be less apparent during binocular fixation. However, those studies analyzed the mean gaze positions of the right and left eyes collectively rather than examining each eye’s individual gaze position during binocular viewing. Additionally, fixation stability could not be assessed during monocular viewing under covered conditions.

Because eye trackers detect fixation using infrared cameras, incorporating an infrared filter could allow for the assessment of fixation stability in both exposed and covered eyes. This approach may make it possible to measure fixation stability under both closed-loop binocular (both eyes open) and open-loop monocular viewing (one eye closed) conditions [26,27].

Fixation stability evaluation can improve patient care or diagnosis of eye fixation disorders related to ocular health. We aimed to evaluate fixation stability in the right and left eyes under three conditions: binocular viewing, gazing (fixating) during monocular viewing, and covered non-fixation during monocular occlusion. We also examined whether fixation stability is related to eye dominance and phoria. These findings provide insight into how fixation stability is influenced by binocular coordination, eye dominance, and phoria, offering valuable implications for both clinical and research settings.

## 2. Materials and Methods

### 2.1. Participants and Clinical Measures

The required minimum sample size was determined using G*Power 3.1 software (version 3.1.9.4; Heinrich-Heine-Universität Düsseldorf, Düsseldorf, Germany). The sample size needed for the Wilcoxon signed-rank test was calculated for the control group based on an effect size of 0.54. This effect size was derived by converting the mean ± standard deviation of log-transformed bivariate contour ellipse area (log_10_BCEA) for binocular and monocular viewing from the reference data [27] (−0.88 ± 0.28 and −0.59 ± 0.26, respectively) into exponent values (0.13 ± 0.18 and 0.26 ± 0.32, respectively). The calculation also incorporated the strongest correlation coefficient (0.663) between binocular and monocular viewing from the control group reference data [28], which were converted into exponent values. With a significance level (α) of 0.05 and statistical power (1-β) of 0.80, the minimum sample size was calculated as 31. In this study, 34 participants were recruited, exceeding the minimum required sample size by 10%.

This study included 34 healthy university students (15 males and 19 females) aged 20–27 years. The inclusion criteria required participants to have non-strabismic binocular vision, no history of refractive surgery, no current or past diagnosis of strabismus, and no use of medications that could affect visual function. This study was approved by the Kangwon National University Institutional Review Board (KWNUIRB-2023-02-007-001) and conducted in accordance with the tenets of the Declaration of Helsinki. Written informed consent was obtained from all participants before their inclusion.

All participants underwent clinical optometric examinations to assess habitual visual correction, with or without spectacles or contact lenses [29]. Distance visual acuity at 5 m ranged from 0.70 logMAR (logarithm of the minimum angle of resolution) (0.2 decimal) to −0.08 logMAR (1.2 decimal). Near visual acuity at 40 cm was within normal limits, with no reported difficulty in performing daily activities such as reading and writing. Distance visual acuity was measured using a projection chart (ACP-8; Topcon, Tokyo, Japan), and the refractive power of corrective ophthalmic and contact lenses was measured using a lens meter (LM-15; Topcon, Tokyo, Japan). Ocular dominance was determined using the hole-in-the-card method. Phoria was assessed using the modified Thorington method with a Bernell muscle imbalance measure (MIM) card (BC/1209; Bernell, Mishawaka, IN, USA) by placing a Maddox rod in front of the right eye. Distance phoria was measured at 5 m, and near phoria was measured at a viewing distance of 55 cm using an MIM card calibrated to 40 cm and then converted. The measured prism diopters were recorded as negative for exophoria and positive for esophoria.

### 2.2. Apparatus and Procedure

Fixation stability was assessed using the Clinical Eye Tracker (version 18.04; Thomson Software Solutions, Hatfield, UK) [22,23,24]. This device is equipped with a 70 Hz remote Tobii Eye Tracker (Tobii EyeX; Tobii Technology, Stockholm, Sweden) and features a display that visually presents gaze positions. A 27-inch LCD monitor (1920 × 1080 pixels; LG, Seoul, Republic of Korea) was used to display gaze targets. Tobii eye trackers use near-infrared spectral light to illuminate the eyes and produce light reflection patterns for each eye. These patterns, combined with advanced image-processing algorithms, enable precise tracking of gaze positions. All participants underwent fixation stability testing during habitual visual correction, with or without spectacles, to minimize the influence of new spectacles. The participants were instructed to fixate on the center of the monitor, positioned 55 cm away, using a chin and forehead rest to minimize head movements.

**Figure 1:**
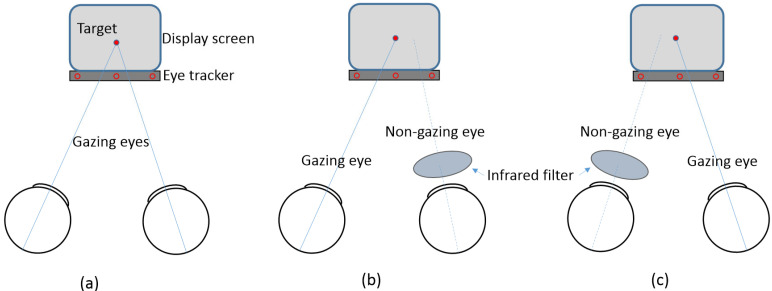
Schematic presentation of viewing conditions. (**a**) Binocular viewing; (**b**) monocular non-fixation for the right eye and monocular viewing for the left eye; (**c**) monocular viewing for the right eye and monocular non-fixation for the left eye.

The eye tracker was calibrated for each participant’s eyes using built-in fixation targets (center, top center, bottom left, and bottom right). Gaze targets consisted of red circular stimuli (3.7 mm diameter, corresponding to approximately 0.66 logMAR) displayed against a black background at the center of the monitor. Participants were asked to fixate on the target for 15 s while measurements were recorded. Gaze data from the monitoring display were recorded as horizontal (*x*-axis) and vertical (*y*-axis) positions, while vergence states were classified as divergence (exo) and convergence (eso). Fixation stability was primarily analyzed based on horizontal and vertical gaze positions. Viewing conditions included binocular, monocular, and monocular non-fixation, as shown in Figure 1. For monocular viewing conditions, one eye was occluded using an infrared filter (Hoya R72 infrared filter; Hoya Corporation, Tokyo, Japan) that blocked light with wavelengths shorter than 720 nm, enabling simultaneous measurement of the viewing and occluded eyes.

Fixation stability data were exported to Excel files for analysis. Data extraction was performed over a 10-s interval, with the start point ranging from 0.5 to 3 s and the end point from 10.5 to 13 s, excluding spark-affected regions within the 15-s measurement period and disregarding eye blinks.

Gaze stability was assessed using the bivariate contour ellipse area (BCEA) [22,23,24,30], which integrates the horizontal and vertical gaze positions according to the following equation:

(1) $$BCEA=2k\pi {\sigma_{h}\sigma_{v}(1-\rho }^{2})$$

where $\sigma_{h}\;$ and $\sigma_{v}$ represent the standard deviations (SD) of the horizontal and vertical gaze positions, respectively, and ρ denotes the Pearson product-moment correlation coefficient between the horizontal and vertical gaze positions. The constant k corresponds to the probability area, where k = 1.146 represents a 68.2% probability area (±1 SD). BCEA was expressed in the pixel area (pixel^2^). To convert the units to degrees (deg^2^), a conversion factor of 0.032°/pixel was applied, based on a reference distance of 55 cm. A smaller BCEA value indicates better gaze stability. If necessary, BCEA plots were generated using the RStudio software (version 1.3.1093; RStudio, Boston, MA, USA).

### 2.3. Statistical Analysis

All statistical analyses were performed using MedCalc (version 12.7.7.0; MedCalc Software, Ostend, Belgium). The D’Agostino-Pearson test was used to determine whether the sample data followed a normal distribution. To compare fixation stability between the eyes under different viewing conditions, the choice of statistical test was based on the normality test result. If the data did not follow a normal distribution, the Wilcoxon signed-rank test or Mann–Whitney U test was applied instead of the paired *t*-test or independent *t*-test. The high and low fixation stability ratios between viewing conditions were analyzed using a chi-square test. The correlation between fixation stability and phoria was assessed using Spearman’s rank correlation coefficient (r_s_). A *p*-value of less than 0.05 was considered statistically significant.

## 3. Results

### 3.1. Participants’ Demographic and Refractive Characteristics

The demographic and refractive characteristics of the 68 eyes of the 34 participants included in the study are presented in Table 1. These characteristics provide insights into the participants’ demographic composition, including age and sex distribution, refractive errors in non-strabismic binocular vision disorders (rather than strabismus), and the degree of ocular deviation, facilitating the interpretation of the study results.

All participants were in their 20s, with a mean age of 22.4 ± 2.1 years. The male-to-female ratio was 15:19, and the number of participants wearing spectacles was twice that of those who did not. Visual acuity below the amblyopia threshold of 0.20 logMAR [7,27] was identified in seven eyes. However, after excluding three eyes with uncorrected refractive errors and one eye with an interocular difference of fewer than two lines, only three eyes (3/68 eyes; 4.4%) were classified as amblyopic, indicating a very low prevalence of amblyopia. Refractive errors were predominantly myopic (67.6%), with no cases of hyperopia. The right eye was dominant in 60.3% of cases (61.8% for distance vision, 58.8% for near vision). Most participants exhibited non-strabismic binocular vision characterized by exophoria at near distances.

### 3.2. Fixation Stability in Binocular and Monocualr Viewing

Fixation stability under three viewing conditions—binocular viewing (closed loop/both eyes open), gazing eye during monocular viewing (open loop), and covered non-gazing eye during monocular viewing (open loop/one eye closed)—was evaluated using BCEA (deg^2^), where lower BCEA values indicate better fixation stability. The median (25th to 75th percentile) and the mean ± standard deviation were as follows: for the right eye in binocular viewing (RE/BV), 0.95 (0.67–1.62) and 1.20 ± 0.87; for the left eye in binocular viewing (LE/BV), 0.75 (0.53–1.22) and 1.02 ± 0.84; for the right eye in monocular viewing (RE/MV), 1.07 (0.72–1.58) and 1.32 ± 0.92; for the left eye in monocular viewing (LE/MV), 0.86 (0.68–1.18) and 1.02 ± 0.58; for the right eye covered in monocular viewing (cRE/MV), 1.62 (1.09–2.30) and 1.87 ± 1.03; and for the left eye covered in monocular viewing (cLE/MV), 1.32 (0.91–2.31) and 1.61 ± 0.96. Across all viewing conditions, the left eye tended to show better fixation stability than the right eye. However, when data from both right and left eyes were combined to increase the sample size, the right eye had a significantly larger BCEA, indicating lower fixation stability (RE vs. LE, Wilcoxon signed-rank test, Z-score = 2.959, *p* = 0.003).

**Figure 2:**
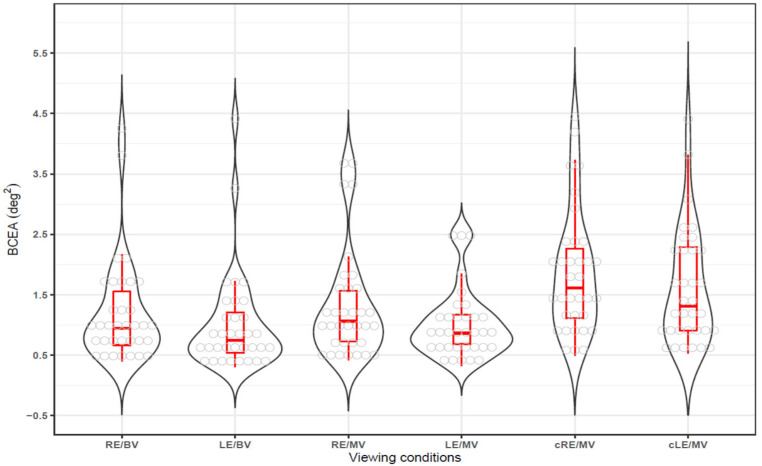
Fixation stability under different viewing conditions. BCEA = bivariate contour ellipse area, RE/BV = right eye in binocular viewing, LE/BV = left eye in binocular viewing, RE/MV = right eye in monocular viewing, LE/MV = left eye in monocular viewing, cRE/MV = right eye covered in monocular viewing, cLE/MV = left eye covered in monocular viewing.

When fixation stability was visualized using box-and-whisker and violin plots, as shown in Figure 2, fixation stability was the highest under binocular viewing conditions (RE/BV and LE/BV), with smaller BCEA values and narrower data distributions. Under monocular viewing conditions (RE/MV and LE/MV), BCEA values increased, indicating slightly reduced fixation stability. For the covered eye conditions (cRE/MV and cLE/MV), the BCEA values increased significantly, showing the lowest fixation stability.

A significant difference in the proportion of high vs. low BCEA values between the right and left eyes observed across viewing conditions (χ^2^(5) = 13.412, *p* = 0.020). In 22 of the 34 cases (64.7%), LE/BV was smaller than RE/BV. Similarly, in 21 of the 34 cases (61.8%), both LE/MV and cLE/MV were smaller than RE/MV and cRE/MV, respectively. However, the difference in fixation stability between right and left eyes was not statistically significant.

As shown in Table 2, a comparison of fixation stability between binocular and monocular viewing showed that the difference in BCEA was statistically significant between the left eye in binocular viewing and the right eye in monocular viewing (LE/BV vs. RE/MV; Wilcoxon signed-rank test, *p* = 0.029), with the left eye demonstrating better fixation stability in binocular viewing. Moderate positive correlations were observed between the right eye during binocular viewing and the right eye during monocular viewing (r_s_ = 0.484, *p* = 0.005), while a weak positive correlation was observed between the left eye during binocular viewing and the left eye during monocular viewing (r_s_ = 0.345, *p* = 0.047).

### 3.3. BCEA in Gazing and Non-Gazing Eyes in Monocualr Viewing

The BCEA values of the gazing and non-gazing eyes during monocular viewing are presented in Table 3. The difference in BCEA between gazing and non-gazing eyes was statistically significant in all comparisons except between the right gazing eye and the left non-gazing eye (RE/MV vs. cLE/MV). All cases showed moderate to strong correlations (r_s_ = 0.401–0.646). However, no significant BCEA differences were found between the right and left eyes for either the gazing or non-gazing conditions. Nonetheless, fixation stability was consistently better in the gazing eye than in the non-gazing eye during monocular viewing.

### 3.4. Comparison of BCEA in Dominant and Non-Dominant Eyes

A comparison of the BCEA between the dominant and non-dominant eyes, which relates ocular dominance to visual functions, such as eye movement [31], is presented in Table 4. Under binocular viewing, monocular viewing with gazing, and monocular viewing without gazing conditions, the non-dominant eye exhibited better fixation stability than the dominant eye in the monocular viewing without gazing condition (Wilcoxon signed-rank test, *p* = 0.008). However, no significant differences were observed under the other conditions.

### 3.5. Comparison of BCEA Between Doinmant Eyes

A comparison of fixation stability according to viewing conditions for the dominant eye (right eye for 20 participants, left eye for 14 participants), is shown in Table 5. Under binocular viewing conditions, BCEA of the right eye differed significantly between right-eye and left-eye dominance (Mann–Whitney test, *p* = 0.046). However, no significant differences were observed under other conditions.

### 3.6. Correction Between BCEA and Phoria

A correlation analysis to investigate the relationship between the degree of phoria, a latent form of eye misalignment [32], and BCEA is presented in Table 6. Under binocular viewing conditions, a significant correlation was observed between the left eye and phoria (r_s_ = 0.413, *p* = 0.018). No significant correlations were found under other conditions.

## 4. Discussion

Fixation stability has primarily been observed with the naked eye in clinical practice. However, an objective and detailed assessment can be conducted using an eye tracker, enabling the detection of subtle eye movements and allowing for the development of a more precise evaluation method. In this study, we investigated fixation stability under three conditions: binocular viewing, monocular viewing with gaze, and monocular viewing without gaze in participants with non-strabismic binocular vision, a key focus in optometry. Fixation stability was generally better during binocular and monocular viewing with gaze than during monocular viewing without gaze. Although no statistically significant differences in fixation stability were observed between the right and left eyes, the left eye tended to exhibit greater stability. When comparing the dominant and non-dominant eyes, fixation stability in the left eye during binocular viewing was higher when the left eye was dominant. In monocular viewing without gaze, the non-dominant eye demonstrated better fixation stability than the dominant eye. Additionally, a significant quantitative correlation was found between fixation stability and the degree of phoria, particularly in the left eye during binocular viewing.

Previous studies on fixation stability assessment [27] suggested using multiple methods due to differences in BCEA, kernel density estimation, and scanpath methods. However, to facilitate comparisons with other studies, this study evaluated the fixation stability using BCEA, a widely applied method.

The results of fixation stability evaluations under the three conditions showed an average BCEA ranging from 1.01 to 1.87 deg^2^, which was higher than the values reported in other studies, such as 0.56 deg^2^ [33] and 0.57 deg^2^ [34]. In contrast, a previous study on subjects with normal phoria reported lower values, ranging from 0.24 to 0.52 deg^2^ [24]. A recent study evaluating monocular non-viewing in patients with amblyopia reported BCEA values in the control group ranging from 0.63 to 1.23 deg^2^ [35]. These differences may be attributed to variations in the study populations [27,34,36], experimental conditions such as fixation targets [37], and measurement devices [13,38]. Most notably, differences were likely influenced by degree of refractive error, binocular vision disorders, and whether the non-gazing eye was assessed.

When comparing fixation stability between binocular and monocular viewing, both eyes generally exhibited better stability in binocular viewing than in monocular viewing [28]. However, in contrast to previous studies, which reported better fixation stability in binocular viewing for both eyes, this study found that only the left eye demonstrated better fixation stability in binocular viewing compared to the right eye in monocular viewing. No other statistically significant differences were observed, although BCEA values for binocular viewing tended to be lower than those for monocular viewing. Contrary to previous findings that reported differences in fixation stability between the amblyopic and fellow eye [7], this study found no statistically significant difference between the right and left eyes. This may be due to the low prevalence of amblyopia (4.4%) among the participants, which likely had minimal impact on fixation stability. Applying these results to closed- and open-loop systems offers an alternative perspective. In both binocular and monocular views (with and without gaze), the eyes maintain retinal image stability through accommodative vergence eye movements [39]. This study confirmed that binocular viewing occurred in a closed-loop condition, while gazing and non-gazing eyes in monocular viewing occurred in an open-loop condition. Under both conditions, both eyes moved simultaneously and the vergence movements of both eyes continued even in the open-loop state. Additionally, in the open-loop condition, the covered eye exhibited larger movements [26].

Fixation stability of the gazing and non-gazing eyes in monocular viewing is consistent with previous research [28], which found that gazing eyes exhibit better fixation stability than non-gazing eyes in monocular viewing. The results correspond to previous findings [40] showing that the BCEA is larger in the covered eye than in the gazing eye under monocular conditions and that the gazing eye exhibits a larger BCEA than either eye under binocular conditions. While the gazing eye continuously followed the target, the covered eye deviated from it, as suggested by prior studies [41]. This supports the conclusion that the fixation stability of the covered eye is lower. However, these results contradict Hering’s law, which states that eye movements are governed by a single neural command. These findings suggest the presence of asymmetric eye movements deviating from Hering’s law [42,43]. A rare vertical movement pattern can occur during a cover test, where one eye remains stationary while the other moves during the transition from covering to uncovering. This temporary disobedience of Hering’s law is followed by realignment once fixation is regained [44].

The frequency of right-eye dominance was 60.3%, which is lower than the reported range of 61.3–71.5% [31,45]. In contrast to previous studies [3], which found no difference in fixation stability between the dominant and non-dominant eyes, this study observed that the non-dominant eye exhibited better fixation stability than the dominant eye in the gazing state. Earlier research [46] found that in 7- and 8-year-olds, fixation in the dominant eye was more stable than that in the non-dominant eye, with no differences observed in older age groups. In this study, monocular fixation stability, as measured by the BCEA, ranged from 1.02 to 1.87 deg^2^, which was greater than the binocular fixation stability values reported in previous studies [24,33], which ranged from 0.24 to 0.52 deg^2^ and 0.8 deg^2^. These findings indicate that fixation stability varies depending on measurement equipment, experimental conditions, and participant characteristics.

When comparing fixation stability in the dominant eye, fixation stability was better when the left eye was dominant. This result is probably due to the possibility that the monocular deviation with changes in fixation distance was larger in the rightward direction than the leftward direction, regardless of the dominant eye’s direction [47]. Moreover, research using the von Graefe method for phoria testing revealed that the right eye showed more exotropic deviations than the left eye [48]. This suggests that the fixation stability of the left eye may be better than that of the right eye. While fixation stability is generally better in the dominant eye than in the non-dominant eye [7,12,35], there is no clear evidence that the right eye is more stable than the left. In this study, the non-dominant and left eyes exhibited better fixation stability than the right dominant eye in individuals with a dominant right eye. According to previous studies [46], fixation is more stable in the dominant eye at a younger age and improves with age, with no significant differences between the two eyes in adulthood. Furthermore, when dominance was not considered, no differences were observed between the right and left eyes. Another study involving adults reported that the non-dominant eye was less stable than the dominant eye [49], while another report found no difference [50].

Phoria showed a distinct quantitative correlation with BCEA of the left eye during binocular viewing. The correlation with the left eye in binocular viewing was measured by positioning the Maddox rod in front of the right eye and measuring phoria, while the left eye remained open during the modified Thorington phoria test. This suggests a relationship between maintaining the gaze in the left eye. This relationship is believed to be associated with horizontal phoria when one eye is occluded, as the apparent position of the subject correlates with phoria [32,51]. In a previous study [22], no correlation was found between fixation stability and phoria when using binocular gaze position, which averaged the gaze positions of the right and left eyes. However, a correlation was observed between the amount of deviation in the gazing plane, both in the front and behind, and abnormal phoria. In this study, a correlation between fixation stability and phoria was observed when the left eye was gazing at the binocular view. This suggests that evaluating the gazing position of each eye separately, rather than using the average binocular gaze, reflects the gazing state more accurately. Therefore, considering the previous findings that fixation stability differences exist between monocular gazing and non-gazing eyes and that there is a correlation between the position of the occluded eye and phoria, it is necessary to reflect both right and left eye occlusion when assessing eye movements or ocular alignment abnormalities during monocular occlusion tests.

This study had some limitations. It focused only on individuals with phoria, excluding those with strabismus, and did not analyze factors that may influence fixation stability, such as visual acuity, refractive power, and binocular vision abnormalities. Additionally, the small sample size and use of non-parametric methods for analysis may limit generalizability. The need for future expansion in terms of clinical application and consideration of diagnostic accuracy should be addressed. However, a previous study [24] showed no significant difference in fixation stability between normal and abnormal groups in binocular vision when comparing the averages before and after blinking. Moreover, of the 68 eyes included in the study, only 4 showed the possibility of amblyopia, suggesting that amblyopia did not significantly impact the results. Despite these limitations, the evaluation and comparison of fixation stability between the right and left eyes in both binocular and monocular views are still feasible.

Fixation stability holds fundamental scientific importance and contributes to a deeper understanding of oculomotor dynamics, binocular coordination, and interactions within the visual system. Furthermore, it offers critical insights for detecting abnormalities associated with motor and neurological disorders, such as Parkinson’s disease [19]. Therefore, objective assessment of fixation stability using eye-tracking technology presents valuable implications for both clinical and research settings. The findings of the present study will enhance clinicians’ comprehension of gaze stability under varying viewing conditions, potentially improving diagnostic accuracy and treatment strategies for patients with gaze-related disorders. For optometrists, this knowledge aids in conducting more precise evaluations, thereby elevating the quality of patient care during eye examinations. As a result, patients benefit from more accurate diagnoses and personalized treatment plans, leading to better management of conditions affecting gaze stability, such as binocular vision disorders or visual instability. In addition, the precision offered by eye tracking could facilitate earlier detection of visual dysfunctions and support the development of tailored therapeutic interventions. This study is currently in an experimental stage; however, it lays the groundwork for future clinical integration, highlighting the potential of fixation stability analysis in routine eye examinations to monitor treatment progress and assess the effectiveness of visual therapies.

## 5. Conclusions

In a study of healthy individuals in their 20s, the fixation stability in non-strabismic eyes, including those with phoria, was measured using eye tracking. The results showed that fixation stability in the left eye during binocular viewing was better than that in the right eye during monocular viewing. During monocular viewing, the gazing eye demonstrated better fixation stability than the non-gazing eye, indicating a trend in fixation stability as follows: binocular viewing, monocular viewing, and non-fixating monocular conditions. However, no significant differences in fixation stability were observed between the right and left eyes across the three conditions.

When comparing the dominant and non-dominant eyes, fixation stability in the left eye during binocular viewing was better when the left eye was dominant than when the right eye was. Similarly, in the non-gazing monocular condition, the non-dominant eye showed better fixation stability than the dominant eye. Furthermore, the link between phoria and fixation stability—depending on which eye is covered—highlights the need to test both eyes separately when checking for issues such as eye movement and ocular alignment in tests that involve occlusion.

## Figures and Tables

**Table 1:** Demographic and refractive characteristics of the study participants.

	Mean ± SD	Range
Male/female (*n*)	15/19	
Age (years)	22.4 ± 2.1	20—27
Refractive correction/no correction (*n*)	23/11	
Decimal visual acuity (logMAR)	0.07 ± 0.16	−0.08—0.70
Better than 0.15/worse than 0.20 (eyes)	61/7	no correction: 3
Refractive errors (*n*)		
Myopia/emmetropia/not confirmed (*n*)	23/6/5	
Corrected refractive power (D, diopters)		
Spherical power	−2.88 ± 1.42	0.25—−5.75
Cylindrical power	−1.24 ± 0.93	−0.25—−3.50
Spherical equivalent	−3.32 ± 1.53	−0.25—−6.50
Phoria (△, prism diopters)		
Distance	−2.24 ± 2.35	1.50—−9.50
Near	−5.91 ± 5.42	1.00—−24.00
Dominant eyes: right/left eyes (*n*)		
Distance	21/13	Distance ≠ near: 3
Near	20/14	

**Table 2:** Comparison of BCEA during binocular and monocular viewing.

	*n*	Wilcoxon Signed-Rank Test		Correlation Coefficient	
		Z-Score	*p*-Value	r_s_^1^	*p*-Value
RE/BV vs. RE/MV	34	−0.563	0.573	0.484	0.005 *
RE/BV vs. LE/MV	34	0.427	0.669	0.007	0.966
LE/BV vs. RE/MV	34	−2.188	0.029 *	0.308	0.077
LE/BV vs. LE/MV	34	−0.241	0.809	0.345	0.047 *

The abbreviations refer to the notes in Figure 2. ^1^ Spearman’s rank correlation coefficient. * *p* < 0.05, significant difference.

**Table 3:** Comparison of BCEA between gazing and non-gazing eyes during monocular viewing.

	*n*	Wilcoxon Signed-Rank Test		Correlation Coefficient	
		Z-Score	*p*-Value	r_s_^1^	*p*-Value
RE/MV vs. cRE/MV	34	−3.274	0.001 *	0.646	<0.001 *
RE/MV vs. cLE/MV	34	−1.903	0.057	0.580	0.001 *
LE/MV vs. cRE/MV	34	−4.197	<0.001 *	0.401	0.021 *
LE/MV vs. cLE/MV	34	−3.830	0.001 *	0.444	0.011 *

The abbreviations refer to the notes in Figure 2. ^1^ Spearman’s rank correlation coefficient. * *p* < 0.05, significant difference.

**Table 4:** Comparison of BCEA between dominant and non-dominant eyes.

	Dominant Eye (*n* = 34)		Non-Dominant Eye (*n* = 34)		Wilcoxon Signed-Rank Test	
	Median	Range	Median	Range	Z-Score	*p*-Value
BV	0.97	0.56–1.62	0.73	0.57–0.99	1.864	0.062
MV	1.01	0.69–1.85	1.06	0.77–1.35	0.060	0.952
cMV	1.65	0.96–2.38	1.11	0.71–2.14	2.653	0.008 *

BV, binocular viewing; MV, monocular viewing; cMV, covered monocular viewing. Range = 25th to 75th percentile. * *p* < 0.05, significant difference.

**Table 5:** Comparison of BCEA between dominant eyes.

	RE Dominant (*n* = 20)		LE Dominant (*n* = 14)		Mann–Whitney U Test	
	Median	Range	Median	Range	Z-Score	*p*-Value
RE/BV	1.12	0.84–1.73	0.73	0.58–0.96	1.995	0.046 *
LE/BV	0.71	0.57–1.26	0.83	0.50–1.22	0.157	0.875
RE/MV	1.17	0.76–1.63	0.97	0.63–1.28	0.980	0.327
LE/MV	0.77	0.65–1.22	0.94	0.77–1.18	−0.490	0.624
cRE/MV	2.03	1.21–2.70	1.41	0.94–1.95	1.505	0.132
cLE/MV	1.26	0.80–2.42	1.44	0.91–1.80	0.192	0.847

The abbreviations refer to the notes in Figure 2. Range = 25th to 75th percentile. * *p* < 0.05, significant difference.

**Table 6:** Correlation between phoria and BCEA.

	r_s_^1^	*p*-Value
RE/BV	0.091	0.600
LE/BV	0.413	0.018 *
RE/MV	−0.020	0.908
LE/MV	−0.075	0.665
cRE/MV	−0.179	0.305
cLE/MV	−0.314	0.072

The abbreviations refer to the notes in Figure 2. ^1^ Spearman’s rank correlation coefficient. * *p* < 0.05, significant difference.

## Data Availability

The data presented in this study are available upon request from the corresponding author.
